# Surgical training in extended sleeve lobectomy with fresh-frozen human cadavers: a case report

**DOI:** 10.1186/s44215-023-00032-7

**Published:** 2023-05-04

**Authors:** Shinichi Sakamoto, Hiromitsu Takizawa, Fuyumi Izaki, Taihei Takeuchi, Hiroyuki Sumitomo, Naoki Miyamoto, Mika Takashima, Naoya Kawakita, Hiroaki Toba, Yoshihiro Tsuruo

**Affiliations:** 1 Department of Thoracic and Endocrine Surgery and Oncology, Institute of Biomedical Sciences, Tokushima University Graduate School, Kuramoto, Tokushima, 770-8503 Japan; 2 Department of Anatomy and Cell Biology, Institute of Biomedical Sciences, Tokushima University Graduate School, Kuramoto, Tokushima, Japan

**Keywords:** Extended sleeve lobectomy, Fresh-frozen human cadavers, Pneumonectomy

## Abstract

**Background:**

Extended sleeve lobectomy is a useful technique for avoiding pneumonectomy; however, it requires advanced surgical skills.

**Case presentation:**

Herein, we report the case of a 67-year-old male, who presented with locally advanced lung adenocarcinoma in the left lower lobe and was scheduled to undergo extended sleeve lobectomy (left lower lobectomy + lingulectomy). In order to complete this operation safely, we practiced the procedure on fresh-frozen human cadavers 2 weeks before the surgery. The cadaveric tissue was soft, and the bronchi exhibited comparable fragility to those in the living body. During the actual surgery, the bronchoplasty procedure matched our experience with the cadaver model, and the patient’s postoperative course was uneventful.

**Conclusions:**

Fresh-frozen human cadavers are useful for training surgeons in technically demanding procedures.

## Background

Pneumonectomy has the highest operative mortality rate of all operations for primary lung cancer [1, 2]. Moreover, it may have a great impact on patients’ postoperative quality of life because it not only causes respiratory function loss but also increases the right heart load due to loss of the pulmonary vascular bed. The main causes of death from pneumonectomy include circulatory complications, acute respiratory distress, acute respiratory distress syndrome, arrhythmia, and renal disease [3]. In fact, it is said that “Pneumonectomy is a disease in itself” [4]. Therefore, surgeons must always carefully consider whether it is possible to avoid pneumonectomy by performing bronchoplastic lobectomy or angioplasty instead.

Extended sleeve lobectomy is defined as an atypical bronchoplasty procedure involving the resection of more than one lobe [5]. It is a feasible procedure that does not compromise oncological principles. However, due to the large degree of variation in bronchial caliber, the fragility of the distal stump, and the increased tension at the anastomotic site, extended sleeve lobectomy is technically more difficult than simple sleeve lobectomy. We report a case in which preoperative simulations of extended sleeve lobectomy were carried out using fresh-frozen human cadavers, which resulted in extended sleeve lobectomy type C (left lower lobectomy + lingulectomy) [4] being performed safely.

## Case presentation

**Fig. 1:**
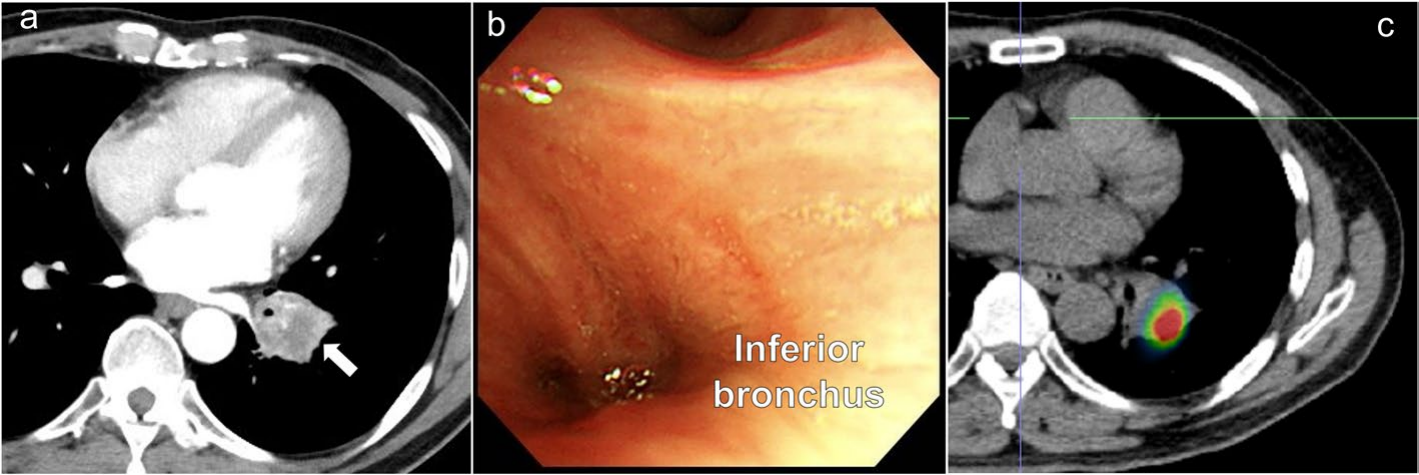
Preoperative chest computed tomography, bronchoscopic, and 18F-fluorodeoxyglucose positron emission tomography-computed tomography images. Chest computed tomography revealed a tumor in the left inferior lobe (white arrow) **a** Bronchoscopy showed that the left inferior bronchus had been occluded by the tumor **b** 18F-fluorodeoxyglucose positron emission tomography-computed tomography revealed an area of abnormal uptake, covering the tumor and the no. 11 lymph nodes **c**

We report the case of a 67-year-old male, who was referred to our hospital with locally advanced lung adenocarcinoma. Chest computed tomography revealed a tumor (maximum diameter: 30 mm) in the left lower pulmonary lobe, which had invaded the S^4+5^ and hilar lymph nodes (no. 11) (Fig. 1a). Bronchoscopy showed that the left inferior bronchus had been occluded by the tumor (Fig. 1b). 18F-fluorodeoxyglucose positron emission tomography-computed tomography revealed an area of abnormal uptake, which covered the tumor and the no. 11 lymph nodes, but not the mediastinal lymph nodes (cT2aN1M0 stage 2B) (Fig. 1c). To secure appropriate surgical margins, the patient was treated with two cycles of carboplatin and tegafur–gimeracil–oteracil potassium plus 40 Gy radiation, which was administered to the hilar lymph nodes (response evaluation criteria in solid tumors: stable disease). His preoperative pulmonary function was as follows: vital capacity (VC): 3100 ml, forced vital capacity (FVC): 3110 ml, forced expiratory volume in one second (FEV1): 2430 ml, and percentage of predicted FEV1 (FEV1%): 94.9%. We planned extended sleeve lobectomy, type C, in order to avoid pneumonectomy. The patient’s predicted pulmonary function after the surgery was as follows: VC: 2070 ml, FVC: 2073 ml, FEV1.0: 1620 ml, and FEV1%: 63.3%.

**Fig. 2:**
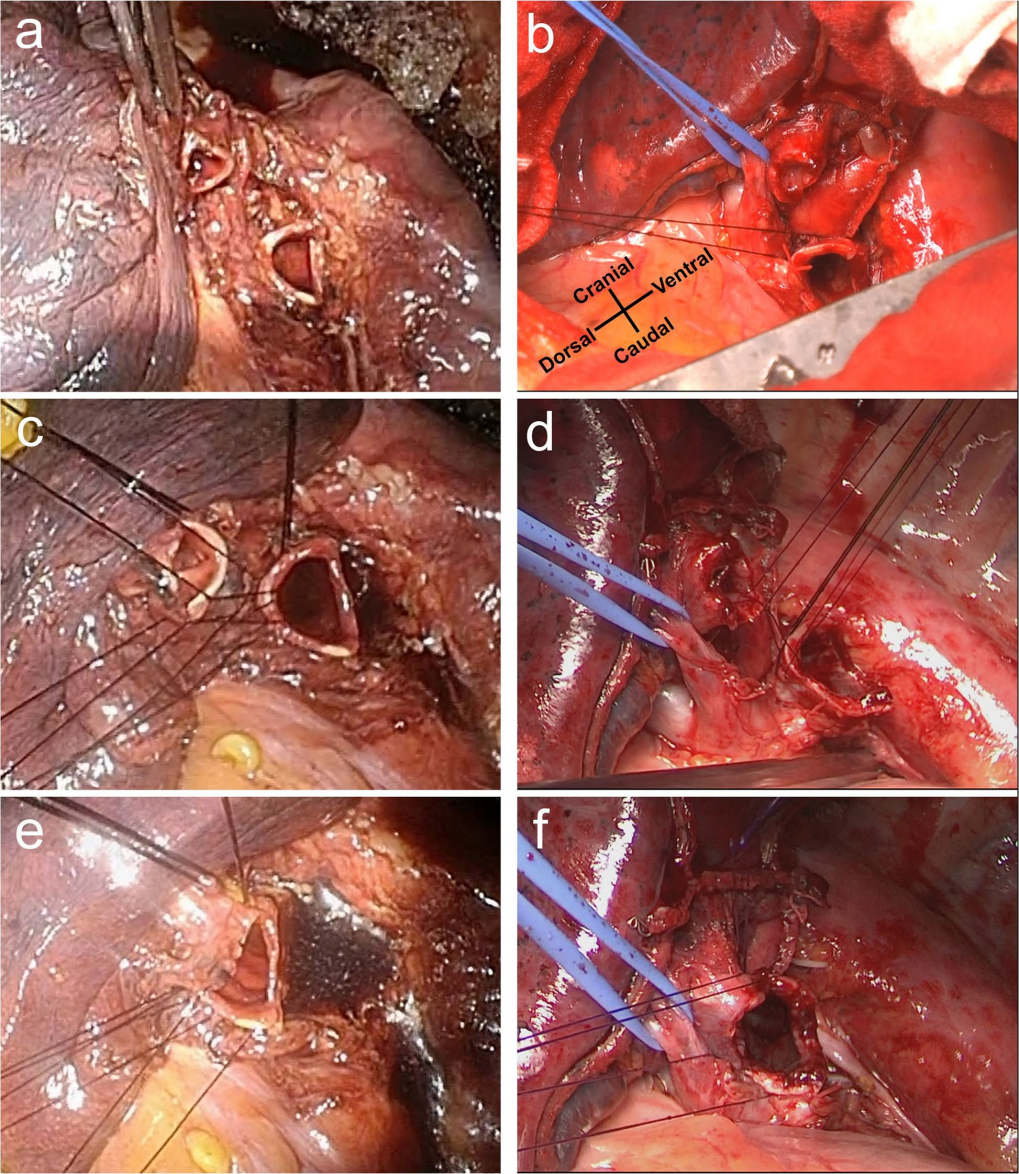
Fresh-frozen human cadaver and intraoperative findings. Fresh-frozen human cadaver findings **a**, **c**, **e** Intraoperative findings **b**, **d**, **f** Images obtained after lower lobectomy and lingulectomy **a**, **b** Three interrupted sutures were inserted in the deep part of the surgical field **c**, **d** Next, further interrupted sutures were inserted, starting from the deepest point of the surgical field and moving in the lateral direction **e**, **f**

**Fig. 3:**
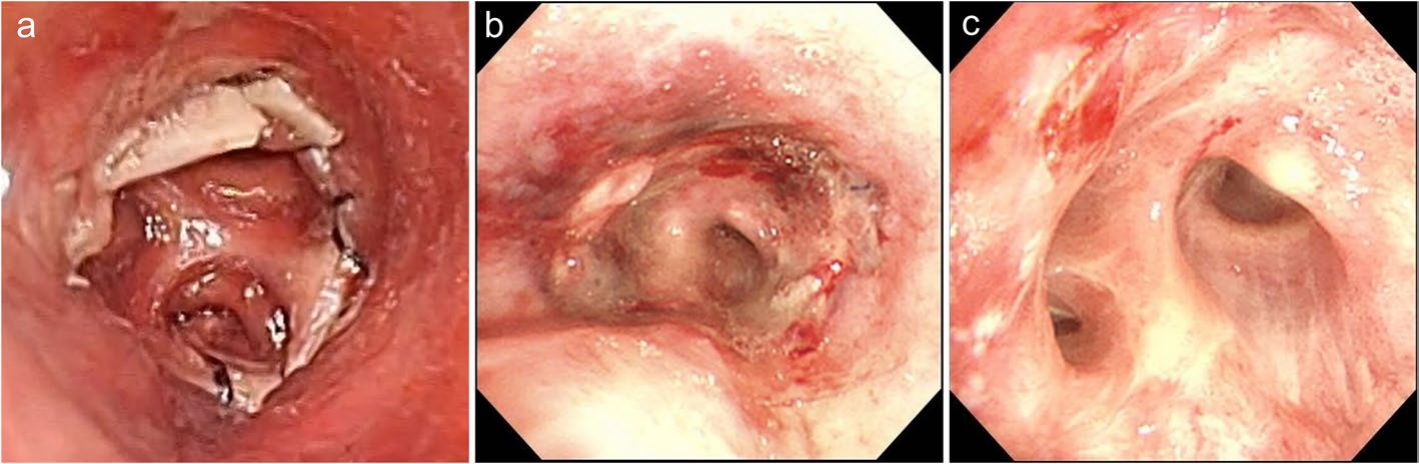
Anastomotic region. After cadaver training **a** One day after surgery **b** Three months after surgery **c**

We decided to perform surgical training using fresh-frozen human cadavers 2 weeks before the surgery. The condition of the cadaveric tissue was soft and comparable to that encountered in the actual surgery, and we performed bronchoplasty after lower lobectomy and lingulectomy on the cadavers (Fig. 2 a and b). The major stages of the bronchoplasty procedure were as follows:

1. We inserted three interrupted sutures in the deep part of the surgical field and tied the middle suture outside the deepest part of the surgical field (Fig. 2 c and d).
2. The next sutures were inserted and tied off, starting from the deepest point of the surgical field and moving in the lateral direction (Fig. 2 e and f).
3. The suturing of the membranous part of the bronchus was adjusted in order to match the proximal and distal bronchial stumps. Lastly, we cut the left main bronchus and checked the anastomosis from inside the trachea (Fig. 3a).

During the actual surgery, we performed a standard posterolateral thoracotomy through the fourth intercostal space. The procedure was the same as for a normal lobectomy, and the mediastinal lymph node dissection was completed before the bronchoplasty. The findings obtained during the bronchoplasty were very similar to those obtained from the fresh-frozen human cadavers (Fig. 2 a–f). We checked the proximal and distal margins and obtained a rapid pathological diagnosis. The pathological examination showed that there had been no invasion into the distal or proximal margins. We used double-armed 4-0 polydioxanone sutures (PDS, Ethicon, Somerville, NJ, USA). The bronchoplasty procedure was the same as that performed on the fresh-frozen human cadavers. A pericardial fat pad was inserted between the bronchial anastomosis and the pulmonary artery. The operation time was 322 min, and 60 ml of intraoperative blood loss occurred. Pathologically, the tumor was diagnosed as an invasive adenocarcinoma with a maximal diameter of 35 mm, and the pathological stage was p-T2aN1M0 stage 2B. The patient’s postoperative course was uneventful, and he was discharged from hospital 8 days after the surgery. Postoperative bronchoscopy revealed a good anastomotic region (Fig. 3 b and c). At 3 postoperative months, the patient’s pulmonary function was as follows: VC: 2420 ml, FVC: 2350 ml, FEV1: 2020 ml, and FEV1%: 79.5%.

## Discussion and conclusions

The operative mortality rate of sleeve lobectomy ranges from 1.2 to 7.5%, while that of pneumonectomy ranges from 4.9 to 12.0%, and the 5-year overall survival (OS) of sleeve lobectomy is better than that of pneumonectomy [3, 5]. The greatest advantage of total pneumonectomy is that it makes it possible to completely dissect the intrapulmonary and hilar lymph nodes, but the locoregional recurrence rates of pneumonectomy and sleeve lobectomy were reported to be 22.8% and 15.6%, respectively (*p* = 0.69) [3]. Therefore, it is generally recommended that pneumonectomy should be avoided, and sleeve lobectomy should be performed instead. There was no significant difference in 5-year OS between extended sleeve lobectomy and simple sleeve lobectomy (62% and 69%, respectively); however, bronchopleural fistulas were significantly more common after extended sleeve lobectomy than after simple sleeve lobectomy, and switching to pneumonectomy due to anastomosis-related complications was significantly more common in extended sleeve lobectomy [5]. Extended sleeve lobectomy involves a more advanced technique, and concerns about the risk of bronchial complications restrict its widespread use. In particular, one of the features of type C extended sleeve lobectomy is that, unlike in the type A and type B procedures, the angle between the left main bronchus and the upper division bronchus is 90°, which may make it difficult to adjust the two stumps [6]. Therefore, in order to complete this operation safely, it is necessary to be familiar with complicated procedures, and a detailed plan is required before the operation. However, the annual frequency of sleeve pneumonectomy in Japan was reported to be 1.0% (474/44859), making it a rare procedure [7], and the chances of experiencing extended sleeve lobectomy are extremely low. This raises a question about how surgeons can be trained in extended sleeve lobectomy.

Practical surgical training is not possible after formalin fixation because the texture, elasticity, joint mobility, and nerve and blood vessel properties of formalin-fixed tissue are significantly different from those of living tissue. However, cadaver training can be performed using the Thiel method or fresh-frozen human cadavers. At Tokushima University Hospital, fresh-frozen human cadavers are used to perform preoperative simulations of demanding operations. This study was approved by the Tokushima University Hospital institutional review board (no. 2246). Physicians from other institutions can use fresh-frozen human cadavers if they attend a seminar and are certified as a registered clinical trial physician at the University of Tokushima.

The use of a fresh-frozen human cadaver has to be supported by the deceased, who donates their body, and their bereaved relatives, who respect the wishes of the deceased. Cadavers are provided for training as soon as possible after death. After it has been confirmed that a cadaver is free from infection, such as by the hepatitis B and C viruses and syphilis, it is stored at −20 °C. There are some issues with the use of fresh-frozen cadavers, including a risk of infection, high maintenance costs, the difficulty of repeatedly freezing and thawing the cadavers, and the limited time in which the cadavers remain in a good condition that is close to the state of the living body. However, we consider that fresh-frozen cadavers were the most suitable model for our study. In the fresh state, the color tone and feel of cadaveric tissues, such as skin, muscles, and nerves, are almost the same as those of the living body. The gripping sensation produced by fresh-frozen cadaver tissue is almost the same as that produced by living tissue, and the fragility of tissues can also be experienced by using cadavers. Therefore, it is said that using fresh-frozen human cadavers is superior to the Thiel method for identifying important organs, blood vessels, and nerves [8]. In fact, in our simulations, the colors of the organs in the thoracic cavity were similar to those seen during surgery, and the tissue was soft. In addition, the bronchial fragility of the cadavers was similar to that experienced during surgery, and the sensations associated with grasping and suturing were almost the same as those experienced during surgery.

Considering anatomical structures from a surgical point of view, using fresh-frozen human cadavers before highly difficult surgery is not only useful for ensuring the safety of the surgery but also contributes to improving surgeons’ techniques. Fresh-frozen human cadavers are useful for training surgeons in demanding surgical procedures.

## Data Availability

Not applicable

## Abbreviations

VC: Vital capacity
FVC: Forced vital capacity
FEV1: Forced expiratory volume in 1 second
FEV1%: Percentage of predicted FEV1
OS: Overall survival
